# Gaming disorder in the ICD-11: the state of the game

**DOI:** 10.1186/s12888-025-07576-8

**Published:** 2025-11-21

**Authors:** Alessandro Musetti, Georgios Floros, Matteo Chiappedi, Vasileios Stavropoulos

**Affiliations:** 1 Department of Humanities, Social Sciences and Cultural Industries, University of Parma, Parma, Italy; 2 2nd Department of Psychiatry, Aristotle University of Thessaloniki, Thessaloniki, Greece; 3 Istituto Dosso Verde, Pavia, Italy; 4 School of Health and Biomedicine, Royal Melbourne Institute of Technology (RMIT), Melbourne, Australia

**Keywords:** Disordered gaming, Diagnosis, Review, World Health Organisation, ICD-11, IGD

## Abstract

Adverse effects on wellbeing associated to the excessive usage of video games have prompted the introduction of Internet Gaming Disorder (IGD) as provisional diagnosis by the American Psychiatric Association [APA] in 2013, and the formal classification of Gaming Disorder (GD) by the World Health Organisation [WHO] in 2019. Despite these milestones, ongoing debate surrounds the diagnostic validity and cross-cultural applicability of these classifications. Consequently, the aims of the present review involve to (i) comparably introduce the WHO and the APA suggested criteria related to disordered gaming, whilst taking into consideration the available psychometric evidence internationally; (ii) illustrate the suggested criteria’s broader strengths and weaknesses and; iii) identify areas of priority for further empirical research to contribute to the available knowledge in the field identify areas of priority for further empirical research to contribute to the available knowledge in the field, whilst concurrently synthesizing the available evidence through the lenses of the recently proposed Cyber-Developmental Framework. With the increasing prevalence of disordered gaming and screen-related addictive behaviours as significant mental health concerns globally, this review highlights the need for enhanced diagnostic precision and greater consistency in assessment methodologies across diverse community, clinical, and national populations.

## Introduction

Digital media use has been interwoven with contemporary living globally, serving diverse everyday needs, including education, employment and entertainment [1]. Web-applications have inevitably proliferated (e.g. different social media specialized in romantic relationships, professional or scholar engagement), and have become more technically advanced to address consumers’ requests and optimize their usage experience [2]. In this context, one of the most popular and debated digital applications is gaming [3–5]. Digital games involve using any screen-enabled device, such as computers, television or handheld devices, to inform interactive platforms, where one or more gamers can play concurrently, aiming to accomplish allocated game-tasks [6]. A great variety of different game genres exists [7, 8]. Aside of being played offline (e.g. console games) or online (i.e. internet games), game genres can be categorized based on whether they involve a single or multiple players (e.g. player versus environment; player versus player), whether they require the gamer to play and develop the role of a character evolving within the game-world (e.g. role-playing gamers), and the specific objectives or missions required for completion [7, 8]. The plethora of types of digital games, which is supported to address the preferences and profiles of different audiences, has been suggested to be one of the main reasons underpinning the spread of gaming world-wide [1]. Technological advancements in the delivery of the gaming experience, such as lifelike graphics and animation, as well as the integration of augmented reality combining real and virtual elements as exemplified by games like Pokémon GO, have significantly contributed to the sector’s growth. Additionally, the implementation of artificial intelligence features allows for the customization of gameplay based on user profiles, a concept referred to as “player adaptive games”. These developments have been key factors driving the ongoing expansion of the gaming industry [9, 1]. Unsurprisingly, recent reports estimate the current digital games market worth at US$282.30bn, with a steady yearly increase of over 8% till 2027 and exceeding US$363.20bn within the coming 3 years [10]. The substantial revenue has led to the introduction and expansion of over 180 in-demand college and university courses focused on game development globally [11]. Aligning with these figures, the proportion of gamers in the global population is envisaged to pick from 16,9% in 2024 to 18,5% in 2027, approximating 1,472.0 m people [10].

## From recreational gaming to Gaming Disorder: history of the concept

The release of the first commercially available videogames dates to the early 1970s. Approximately 10 years later, Ross, Finestone and Lavin published the first reports of subjects being over-involved in gaming to an extent which had relevant negative consequences for their lives [12]. This could be considered the beginning of the scientific literature on what the Eleventh Edition of the International Classification of Diseases (ICD) terms “Gaming Disorder” (GD) [13]. In that line, when the fifth edition of the Diagnostic and Statistical Manual of Mental Disorders (DSM-5) was published in 2013, the American Psychiatric Association (APA) decided to include Internet Gaming Disorder (IGD) as a tentative diagnostic category [14]. Diagnostic criteria were provided to stimulate further research on the validity of this construct. Two years later, in 2015, the World Health Organization [WHO] included GD in the beta draft of the eleventh edition of the International Classification of Diseases (ICD-11). This was however only the starting point of a scientific debate: the majority of psychiatrists and psychologists with a clinical orientation supported the adoption of this diagnostic category [15], while others (mainly stemming from the media psychology field) argued against it [16]. They supported that the scientific basis for GD was too weak to be formally listed as a mental health disorder [16]. It is worth noting however, that this decision did not come unexpected: a series of annual WHO expert meetings [including those in Tokyo (Japan), Seoul (South Korea), Hong Kong (China) and Istanbul (Turkey)], held since 2014, had progressively provided a rationale for the recommendation to include GD in the ICD-11 beta draft. Overall, 66 experts from 25 countries took part in these meetings, with strict managing rules and regulations on potential conflict of interest.

On May 25, 2019, the 72nd World Health Assembly of the World Health Organization officially recognized Gaming Disorder as a mental health disorder. This decision faced opposition from the gaming industry [17]. The Entertainment Software Association [ESA] (2019) stated that “the World Health Organization knows that common sense and objective research prove video games are not addictive (…) We strongly encourage WHO to reverse direction on its proposed action” [17], and similar concerns were echoed by the European Games Developer Federation [18]. While gaming is widely acknowledged to offer psychological and even therapeutic benefits [19], this does not negate the reality that a subset of individuals may experience significant harm from excessive gaming behaviours [20]. In response, scholars have advocated for collaboration between the gaming industry and mental health researchers to leverage big data for distinguishing disordered from healthy gaming patterns [21].

In the light of such discussions, ICD-11 classifies Gaming Disorder under “Disorders due to addictive behaviours”, a category rooted in Isaac Marks concept of non-chemical addictions [22]. The diagnosis requires a persistent pattern of gaming behaviour characterised by impaired control (in terms of onset, frequency, intensity, duration, termination, context), prioritisation of gaming over other activities, and continuation or escalation despite negative consequences. A 12-month duration is generally required, though this may be shortened to 6 months in severe cases. Differential diagnoses (e.g., manic episodes, substance use) must be ruled out, and the behaviour must cause significant distress or impairment in personal, family, social, educational, occupational functioning. Co-occurring conditions such as attention deficit hyperactivity disorder, mood, or anxiety disorders are frequently observed [15, 21]. Importantly, the diagnosis should not rely solely on frequent gaming in the absence of other defining features [15, 21]. Although some individuals may report cravings or urges to game, these are considered supplementary rather than core diagnostic criteria.

## Problems with the definition of Gaming Disorder in light of the Cyber-Developmental Framework

**Fig. 1:**
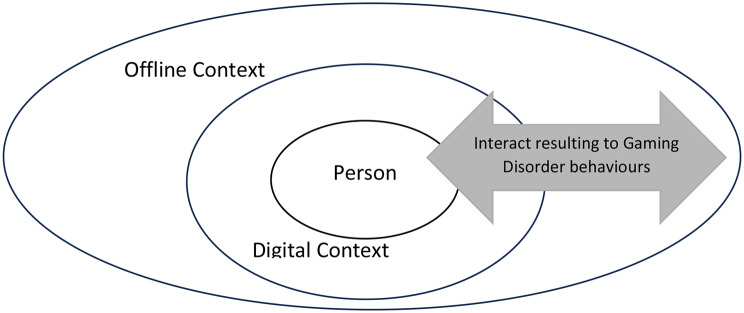
Gaming Disorder through the lenses of the Cyber-Developmental Framework. The Cyber-Developmental Framework (CDF) shows how the intensity of Gaming Disorder behaviours experienced by an individual at a certain point of their development can derive from the interplay between Person (e.g. their specific biological and psychological features), Digital Context (e.g. their cyber-context and activity) and Offline Context (e.g. proximate and distant context)

Following such important developments, there is a sufficient consensus regarding the multiplicity of the aetiology of Gaming Disorder behaviours, as well as their dimensional nature (i.e. problem symptoms can be experienced between a minimum to maximum range likely varying over time [1]). The recently proposed Cyber-Developmental Framework (CDF) implies in particular that, the intensity of Gaming Disorder behaviours experienced by an individual at a certain point of their development, constitutes the byproduct of the interplay between person (e.g. their specific biological and psychological features), digitally contextual (e.g. their cyber-context and activity) and contextual (e.g. proximate and distant context) related effects over time [1]. Figure 1 provides a graphical representation of the CDF model. These sources of influence should be considered for better capturing the GD phenomenology as well as the academic debate related to problematic gaming, currently evolving across four major distinct areas [1, 21]: (A) Differences between the ICD-11 and the DSM-5 disordered gaming classification proposals (person and context level considerations); (B) Construct validity concerns (person and context levels considerations); (C) Defining the fine line between healthy and pathological gaming (person level considerations) and; (D) Group differences in gaming behaviours and disordered gaming risk (person, digital context and contextual levels considerations) [1, 3, 13–16, 19–21].

### ICD-11 Gaming Disorder versus DSM-5 Internet Gaming Disorder (person and contextual considerations)

A recent paper by Yen, Chou, Liao and Ko compared the concepts of IGD in DSM-5 and GD in ICD-11 [23]. The first relevant difference identified, although briefly addressed in their work, referred to the approach adopted to introduce the two constructs. Specifically, while the DSM-5 seemed to aim more towards establishing a clear behavioural difference between “normality” and pathology, the ICD-11 focused more on impairment due to over-gaming, as a necessary diagnostic feature. Non-surprisingly, GD presented with a higher threshold for diagnosis, mainly as a consequence of the rather stronger emphasis on the impairment criterion [23]. The authors also evidenced the importance of correctly differentiating individuals with GD from highly engaged gamers [23]. Moreover, they stressed that the ICD-11 criteria did not include tolerance or withdrawal-like symptoms, although the concept of loss of control on gaming behaviours could be inclusive of such behaviours to some extent [23]. Deception and escapism, considered in DSM-5 as diagnostic criteria, are also not mentioned in ICD-11 [24]. Nevertheless, it has been supported that these may have low diagnostic utility due to being also common features among highly engaged gamers without addiction [24]. Therefore, the ICD-11 diagnosis of GD may bear the potential to reduce the number of both false positives and false negatives compared to the DSM-5 IGD classification [25]. However, the same feature may simultaneously result in failing to identify pertinent issues [26].

A different comparative methodology was followed by Adamkovic et al. [27]. They studied culturally diverse samples of 2,846 young adults engaging in digital gaming and 746 subjects playing eSports. A network approach was applied to explore a multi-verse of GD symptom structures, effects of item operationalization, and possible external moderators. They found that two symptoms (loss of control and continued use despite problems) were systematically central to most of the analysed networks, whilst alternative operationalizations of single items systematically caused significant network differences [27]. Importantly, group variations related to different play styles, age, gender, gaming time, and most of the psychosocial characteristics produced no significant network changes (i.e. network invariance) [27]. In line with this work, King and his colleagues proposed to improve screening tools for GD using alternative (and possibly more stringent) item response categories [28]. Reinforced by such evidence, scholars supported that further validation of empirical studies is required [21, 27, 28]. In particular, the motivational and psychodynamic perspectives offered by Schimmenti et al. [29, 30] provide a compelling complement to these structural critiques. These authors argue that disordered gaming may often function as a compensatory strategy for unmet psychological needs or unresolved trauma, rather than as a stand-alone addiction.

### Construct validity concerns (person and contextual considerations)

In an international Delphi study, Castro-Calvo and colleagues [25] investigated expert agreement on the validity (i.e. the extent to which a criterion is helpful in discriminating adaptive from problematic behaviour) and the utility (i.e. the possibility of a criterion to predict the evolution of the condition) of the disordered gaming criteria. Interestingly, the only criterion from the ICD-11 definition which failed to reach an agreement on its validity was the reduction in interest in non-gaming pursuits, likely due to often reflecting variability, normally distributed to the general population [25]. The fact that further empirical work is still required, is probably emphasized more clearly by the recent findings of Hong et al. [31]. They showed that although the rate of GD cases may not differ significantly at baseline and prospectively, a significant proportion of diagnosed participants (60.4%) had altered diagnosis after a year, with ”Loss of interest” (60.7% changing) and “Loss of Control” (55.7%) presenting to be the least stable diagnostic criteria. As the authors noted, the inclusion of a large proportion of less severe GD clinical presentations in their sample could have contributed to these findings [31]. It was argued that such individuals may frequently have a more variable evolution of their condition (i.e. prognosis) [31]. Moreover, given the significant role that seemed to be played by comorbid features of Attention Deficit Hyperactivity Disorder, the same scholars underlined the importance to have more stringent guidelines on functional impairment evaluations [31], as additionally noted by Billieux et al. [32]. In line with the above, the importance of the amount of time devoted to gaming according to ICD-11 criteria, when compared with functional consequences, is low. This is highly relevant, as the amount of time in one’s life devoted to gaming should include not only time directly devoted to gaming, but also time indirectly consumed by activities and thoughts connected with gaming during “gaming free” intervals (e.g. looking for information to improve gaming performance or discussing about gaming experiences and strategies in person or online) [33].

A rather more critical position regarding the validity of the GD diagnosis was adopted by Ferguson et al. [3]. They studied a sample of 3034 adolescents from Singapore and concluded that, according to their Structural Equation Model, symptoms of problematic gaming were more understandable as a consequence of other mental disorders (notably Mood Disorders and Attention Deficit Hyperactivity Disorder) and not as a distinct condition itself [3]. Therefore, they supported that these should be better viewed as a behavioural attempt to cope with other psychopathological aspects (most notably, low mood; i.e. self-medication hypothesis). To clarify such discrepancies, alike most other psychiatric disorders, the possibility of: (a) biological markers differentiating affected subjects from healthy ones and; (b) distinct behavioural features of GD sufferers could operate as fundamental proofs of the validity of the GD diagnosis and a counterargument to Ferguson and colleagues’ concerns [3, 34].

#### Neurobiological markers

As stated by Vaccaro and Potenza [34], relevant research data is often difficult to pool together due to methodological heterogeneity. Despite this limitation, the majority of such available datasets examined in the international literature seem to identify overlaps in neural activity and cognitive functioning, suggesting that GD cases share similarities, which indeed resemble gambling and substance-use disorders [34]. Indicatively, a posterior parietal activation, specific towards gaming cues has been demonstrated [35]; interestingly, the same activation was not elicitable in subjects without GD even after time-prolonged gaming involvement [35]. Similarly, morphological and functional brain studies have consistently demonstrated the involvement in GD of the reward system [36]; as with other conditions, however, alterations of one or more connectomes, with interest converging on fronto-striatal and subcortical regions and pathways, have been supported to potentially provide more information about GD, than alterations of a single area of the Central Nervous System [37]. For instance, connectivity differences between the lentiform nucleus and the anterior cingulate cortex have been shown to be related to gaming craving following gaming-cues, whilst also being reversible after successful GD treatment [38]. Although such findings regarding the reward system brain regions and pathways commonly implicated with the development of addictions reinforce the addictive classification of the syndrome, further research has been invited to support the construct validity of GD in relation to the biological mechanisms underlying its development, the effect of GD treatment, as well as neurocognitive differences between GD sufferers and non-problematic gamers [34].

#### Behavioural features

In conjunction with such neurobiological evidence, the potential addictive nature of gaming, and to an extent the construct validity of GD, can be further supported by distinct behavioural features such as those corresponding with the components model of addiction [39]. According to this model, the syndrome shares with other behavioural and substance-related addictions six core components: (1) tolerance (i.e. increasing time spent on gaming to achieve previous effects, e.g. to maintain a comparable gaming experience), (2) salience (i.e. gaming becomes an important part of the gamer’s life and plays a dominant cognitive, affective and behavioural role), (3) mood modification (i.e. the use of gaming to influence one’s mood), (4) relapse (i.e. the tendency to revert to previous maladaptive pattern of gaming behaviours, despite attempts to reduce or discontinue it), (5) withdrawal (i.e. unpleasant moods or aversive physiological experience when gaming activity is stopped or reduced), and (6) conflict (i.e. intrapersonal and interpersonal conflicts related to gaming activity).

At this point it should be noted that since all behavioural addictions share a number of core features, differential diagnosis could be challenging if their boundaries are not clearly demarcated (i.e. although an addictive disorder could be clearly identified, determining the exact nature of it could be challenging if multiple addictive symptoms, such as gambling and gaming, are simultaneously present [40]). Reinforcing such concerns, in a recent review of published material in the diagnosis of bipolar disorder and how it relates to GD and Gambling Disorder, Floros and Mylona [40] pointed out that the ICD-11 formulations of GD and Gambling Disorder are essentially identical, with the set of criteria for Gambling Disorder carrying over to GD in their entirety, without a clear justification or prior research data supporting this practice. This may lead to high sensitivity but low specificity in the two diagnoses, with the sole point of differentiation being that a patient with Gambling Disorder jeopardizes a monetary asset for some sort of gain. This is problematic, bearing in mind that a patient with GD typically jeopardizes something of value (personal time, money), while frequently over-gaming in order to attain something of monetary (e.g. tournament prizes, social media followers and advertising) or emotional (internet fame, sense of achievement) value. Thus, one could suggest that supporting the construct validity of GD, as well as the differential diagnosis between Gambling Disorder and GD may be challenging, when features of these two similar phenomenologies are concurrently present.

Since a correct diagnosis can guide a better-tailored intervention [41], the difficulties in the process of differential diagnosis need to be overcome. To help in this respect, there has been progress in both the number and the sophistication of the GD diagnostic tools available, as well as their translation and standardisation across different cultures and languages, taking concurrently into consideration item-response property parameters [28]. In that context, scholars appear to support that more work is needed [28] and engage in a promising WHO Collaborative Project on the Development of New International Screening and Diagnostic Instruments for Gaming Disorder and Gambling Disorder [42].

The comprehensive analysis of such psychometric progress in the light of the scientific debate regarding the validity of the GD diagnosis goes beyond the scope of this narrative review, and as noted by Brand and Potenza [43] the debate “is anticipated to continue for some time”. However, the increasing number of studies on this topic is a proof of the vitality of the discussion, with a progression from an attempt to discuss logical fallacies in other researchers’ opinions to a focus on empirical data [43]. The same presents to apply when it comes to distinguishing healthy from pathologic gaming.

### Adaptive vs. pathological involvement in video games (person considerations)

Specifically, over the past decades, an intense debate among scholars has focused on how to diagnose problematic involvement in video games without pathologizing other forms of gaming engagement, such as playing as a hobby and even passionate, yet non-problematic, use of video games [44, 45]. For instance, the IGD criteria proposed by the APA in the 5th Edition of the DSM-5 [14], conceptualised on the basis of gambling disorder and substance use disorders criteria, have been the subject of much discussion with respect to their actual clinical utility [46]. In fact, this so-called “confirmatory approach” [47] has been suggested to a priori define potentially problematic behaviours (e.g., excessive gaming) as genuine addictions, thus likely failing to capture the specific and unique characteristics of each condition. Accordingly, previous studies have shown that applying substance use disorders criteria (e.g., tolerance and salience) to problematic gaming may result in overdiagnosis [48–50]. Thus, several studies supported the distinction between peripheral (e.g., tolerance, and cognitive salience) and core (e.g., conflict, and withdrawal) symptoms of problematic gaming [51–53]. Indeed, many gamers display peripheral symptoms of disordered gaming without developing a pathological involvement in video games [25, 54]. For example, tolerance can be associated with a variety of reasons explaining why a player increases the time spent on video games, such as contextual events or pleasure associated with gaming [55]. In that line, Deleuze et al. [50] found that the majority of regular gamers (77.3%) endorsed the cognitive salience criterion in their sample. Therefore, it has been deemed as critically important to identify the specific characteristics of pathologic involvement in video games and its boundaries with healthy, yet elevated, involvement to in turn improve GD assessment accuracy and treatment efficacy [48, 56].

Previous research has shown that elevated involvement in video games (as measured by hours of video game use per day or week) is not necessarily associated with maladaptive psychological and social functioning [57, 58]. Based on this evidence, Billieux and colleagues’ review [44] introduced the distinction between “high involvement” (i.e., elevated but healthy use) and “pathological involvement” in video games, which has deeply influenced subsequent research [45]. For example, Musetti et al. [45] adopting a person-centered approach identified four subtypes of online gamers (i.e., occasional, passionate, preoccupied, and disordered gamers), while also taking into consideration time spent playing video games and problematic gaming scores. Their work additionally indicated that non-problematic gamers (i.e. occasional and passionate gamers) represented the majority of the sample [45]. Most importantly, they revealed that passionate gamers showed low levels of psychological maladjustment regardless of the large amount of time consumed on playing video games (i.e. more than 4 h per day). This indicates that the amount of time spent gaming alone does not define disordered gaming, helping to reduce stigma around the activity [45]. Moreover, Musetti et al. [45] identified heterogeneity within the broader group of gamers, portraying two other dominant clusters characterised by low (preoccupied gamers) vs. high (disordered gamers) involvement in video games. Interestingly, preoccupied gamers reported high levels of negative affect (anxiety and depression symptoms) despite not spending excessive amounts of time on video games. This observation aligns with the compensatory internet use framework [59] which posits that problematic online behaviours, such as disordered gaming, may serve as maladaptive coping strategies to compensate for unattained psychosocial needs or alleviate aversive moods [60–62]. This notion reinforces the possibility that problematic gaming behaviours unfold on a continuum (i.e. from minimum to maximum symptoms) and that drawing a definitive line between healthy and pathological use may be conceptually challenging, as multiple factors, and not just time-engagement, need to be considered [63]. Overall, disordered gamers show a pattern of game behaviour characterised by excessive use of video games (more than 6 h per day), addictive-like symptoms and the highest level of impairment in psychological functioning [45]. This finding highlights that high involvement in video games may become maladaptive when it is uncontrolled and inflexible, enhances addictive behaviours and interferes with daily life functioning [64, 65].

Some ambiguous findings on neural correlates of problematic gaming further contributed to doubts about the ability of the DSM-5 criteria to distinguish between high and pathological involvement in video games. For example, a study by Dong et al. [66] showed that regional brain features and communication between brain regions did not significantly differ between gamers who endorsed the DSM-5 threshold for IGD and regular gamers. Such accumulated evidence has suggested the need for more stringent and conservative criteria for pathological gaming [25]. Along these lines, the more recent definition of GD included in the ICD-11 (WHO, 2019), did not include “peripheral” disordered gaming criteria (e.g., tolerance, salience), which may though characterize different subtypes of highly involved gamers. Rather, as previously explained the ICD-11 conservatively chose to give emphasis on functional impairment as a key criterion for distinguishing between high and pathologic involvement in video games (i.e., “gaming pattern must be associated with distress or significant impairment in personal, family, social, or other important areas of functioning”) [44]. Such discrepancies appear to be relevant to the variability occurring among gamers.

### Group differences in gaming engagement and disordered gaming risk (person, digital context and contextual considerations)

There is a number of well-documented differences in the prevalence of disordered gaming within subpopulations of the wider gaming community [1]. For instance, gender has been identified as such a differentiating feature on a meta-analysis by Su et al. [67]. They focused on articles published between January 2010 to August 2019 and found men being: (a) more likely to exhibit disordered gaming behaviours compared to women and; (b) less likely to exhibit social media addiction than women. These differences may be attributed to male-oriented social drives for achievement and greater use of digital devices, contrasted with females’ stronger tendencies toward social expression and orientation [1]. In addition, other, more subtle differences among gamers, that may not be easily discernible or researched, have been implied [1, 21, 67]. Indeed, the heterogeneity of the gaming community with regards to ethnic boundaries has been advocated to potentially generate different engagement pathways, as well as differentiations in one’s gaming experience (e.g. drives for belonging into the player’s in game group for more collectivistic cultures vs. achievement and ranking motivations for more individualistic cultures explained below) [1]. Not surprisingly, the well-known Massively Multiplayer Role Playing Game [MMORPG], World of Warcraft, being highly popular since late 2004, presents with a particularly diverse, contemporary, ethnic player makeup (as per active daily participation), inclusive of individuals from the United States, Russia, Germany, France, the United Kingdom and China (with the latter constituting as many as half of the total number of its players) [68]. The diverse game-engagement mechanics offered by the MMORPG game genre, ranging from online socialization, achievement-challenge to character development, could explain its enduring, multicultural appeal [1]. Overall, population characteristics interfering with every aspect of online gaming, such as regional and country differences in relation to game accessibility, infrastructure and the local culture shaping motives and habits, have been suggested to influence gaming involvement, as well as the optimum disordered gaming assessment practice [1]. It is worth noting that to date, and partially due to such group differences, there is still no gold-standard tool for assessing disordered gaming, although a number of instruments have demonstrated solid psychometric properties and have been employed internationally [69]. Among these, the most commonly used are the Assessment of Internet and Computer Addiction Scale-Gaming [70], the Game Addiction Scale-7 [71], the Ten-Item Internet Gaming Disorder Test [72], the Internet Gaming Disorder Scale-9 Short-Form [73], and the Lemmens Internet Gaming Disorder Scale-9 [74]. For instance, research on the applicability of disordered gaming measures (i.e. the Internet Gaming Disorder Scale–Short-Form 9 items; IGDS9-SF) on gamers hailing from the United States, the United Kingdom and India demonstrated that for the same level of problematic gaming (i.e. latent disordered gaming trait), respondents would rate a different number across the scale items, depending on their origin [75]. Similar findings supporting the need of appropriate modification of disordered gaming scales to address different populations were repeatedly reported by other measurement invariance studies, even among populations sharing the same language [76].

Indeed, the physical locations where gamers engage in play seem to influence the local casual gaming and eSports landscape in distinct ways, as well as to moderate their vulnerability to disordered gaming [1, 77]. For instance, gaming infrastructure, investment and availability in countries where technological advancement may present as a policy priority like South Korea versus countries with more technological hurdles like India, and variations in the official policies regarding gaming in net cafes versus mobile games in China, appear to co-inform how people will choose to game online, their basic level of access to gaming devices, their playing environment and their preferred collective and/or individual gaming engagement [1, 77]. In this example, individual gamers may socialize differently offline due to the game they play depending on the size of their relevant local gaming scene, the size of their country of origin and whether local legislation allows them to meet up to play in public venues or solely online [1]. Accordingly, when examining disordered gaming, demographic factors of interest include not only an individual’s country of origin but also their preferred platform and device for connecting, the scale of their local gaming community (specifically for their chosen game or application), and the degree of their in-person connections with other gamers [1, 21]. A gamer may have to overcome considerably more hurdles in order to game in different environments and those hurdles may render certain disordered gaming criteria as less relevant [13, 14]. For example, a gamer who is by official policy unable to log in for extended periods of time may by definition be less likely to satisfy the tolerance criterion (i.e. DSM-5), while their own sense of control (i.e. ICD-11) may not be challenged since external control revokes their own agency [13, 14]. Additionally, individuals who dedicate substantial effort and resources to gaming in regions with lower income and higher technology costs may face different circumstances than those for whom gaming expenses are minimal relative to income. When conducting studies involving gamers from diverse regions but engaged in the same application, it is necessary to account for these differences by controlling for or assessing their effects using measures such as time spent gaming or financial commitment [1]. Future approaches to assessing problematic gaming could benefit from evaluating the proportion of gaming opportunities utilized, and whether individuals attempt to circumvent external restrictions or would be inclined to do so given favourable circumstances [1, 13, 14, 21, 78].

Aside of such considerations, the interaction of cultural variables of interest with gaming patterns presents to be even more complex [1]. Cultural differences may factor in both the way individuals experience their online gaming world or their real life [1]. One indicative pair of such variables are individualism and collectivism [1, 78]. Although initially defined as cultural syndrome(s), their definition was later expanded to describe individual behaviours by Triandis [78]. People who adhere to their dominant group or community values (i.e. collectivist cultures), compared to people who adhere to their dominant, individually distinct, values (i.e. individualist culture), are likely to define themselves differently based on their group membership, prioritize or not in-group vs. individual goals, be self-effacing, focus on the context (e.g. where the communication takes place or who communicates to them) rather than the content (e.g. what is communicated) of communication and make varying situational attributions [1, 77]. The relation between these individualistic and collectivistic cultural values is yet not dichotomous, with a person being likely differently inclined to behave more individualistically or collectivistically depending on the situation [i.e. continuum from individualism to collectivism, upon which one changes places over their life-course [77]). The default view of East Asian countries is one of having predominantly collectivist cultural syndromes versus Western countries who heavily lean towards more individualistic cultural syndromes [79]. Cultural syndromes can also differ along the horizontal-vertical axis, which describes within-group relationships; ‘horizontal’ indicates the absence of hierarchy or ranking within a group, while ‘vertical’ denotes the presence of hierarchical relationships or inequality [77]. An early study by Lee et al. [80] on social network games found cultural orientations predicted social expected outcomes, which, in turn, mediated cultural effects on game usage patterns. The researchers evaluated social expected outcomes (the motivation to play based on anticipated interactions with others, such as belonging to an in-game group or team) and status expected outcomes (the motivation to play based on anticipated social recognition, such as in-game achievements, rankings, or gains). Collective culture orientations predicted social expected outcomes while individualism predicted status expected outcomes, but in different directions on the dimensions of verticality or horizontality [80]. Two more recent investigations of how these concepts relate specifically to disordered gaming [81, 82] confirmed the differences on collectivism, with gamers who are less vertically collectivistic being more likely to present with greater disordered gaming symptoms.

In addition to an individual’s cultural context and values external to gaming, another significant factor affecting gaming behaviours and the risk of disordered gaming may be the various microcultures that have developed around playing specific games or game genres [1, 83]. These microcultures may be as diverse between them as they are within. Shifts in the dominant culture within can be provoked by factors outside the reach of the community itself, most frequently those imposed by the game developers. An example of note are the tidal waves in the gaming community brought about by the advent of the free-to-play model (F2P), which rapidly became dominant in the gaming titles that garner the most attention. Recent ethnographic research by Elmezeny [83] described in detail those very changes that were brought about both within the gaming culture and the perception of gaming within media and public discourse. The impact in gaming microcultures can readily overspill in real life: tens of thousands of Korean players mobilized in both online and offline protests during Spring 2021, rallying against what they perceived as the gaming’s industry exploitative practices (the ‘Maple refugee’ incident) leading to the adoption of national law to address their concerns [84]. In this event the actual way the game developers framed the laws governing the workings of the game, which directly impacted the community, was in direct conflict with the predominantly skill-oriented culture of the community, leading to a revolt [84]. This is a notable example of how gaming (and overindulgence in gaming) should also be researched from the aspect of the dominant culture within the gaming community. Early studies where players of MMOs took part showed that addictive gaming was not associated with seeking out socialization within the community [85] nor did it link to a negative effect on well-being [86] but rather forming strong social ties to persistent groups which focus on competitiveness or is playing with the self-centred goal of improving one’s own mood [87].

The aforementioned differences may be difficult to fully address in a manner that would be satisfactory, while also providing useful tools for more inclusive, multinational or multicultural comparisons [1]. Indeed, there is a fine line between emic (i.e. culturally specific) and etic (i.e. universally applicable) approaches in designing research and diagnostic tools that would either be more suitable to cater to the needs of a specific population versus employing tools that would be effective (to a large degree) across broader groups. An emic approach maybe ideal if attempting to take into account the dominant characteristics of a gaming microculture (e.g. different levels of research into gaming communities should be carefully defined beforehand, i.e. a study of a subpopulation of gamers from a particular geographical area, playing a genre and being part of the dominant microculture within the genre) [1]. Gaming communities could be well-suited for this kind of culturally specific research since it is far easier to reach more participants than a comparable offline non-gaming community. In this context, the notion and perception of addiction within those gaming communities is an important research topic, since it could lead to the development of outreach or even in-game programs to assist with prevention and early intervention. On the contrary, etic approaches could likely bear the risk of being perceived as alien and potentially hostile, carrying the implicit threat of throwing a bad light to the community, particularly since the official acceptance of GD as a mental disorder.

## Gaming structure & immersion

Despite such inconsistencies regarding the validity and optimum definition of disordered gaming [43], a significant body of literature investigating immersive game features have consistently concluded that the appeal of games is based on their capacity to generate meaningful experiences to their consumers [1, 88–90]. Beyond the context of gaming, research has indicated that individuals are more likely to engage in activities when these: (a) present challenges that encourage them to move beyond their comfort zones (e.g., progressively undertaking more difficult tasks); (b) offer a clear sense of purpose and direction (such as striving towards specific life goals or positions); (c) foster a sense of belonging (for example, through group participation); and (d) support the development of a personal narrative that cultivates confidence and pride [1, 88–90]. These channels of meaning have been supported to be achieved by different structural game features, including the experience of gaming flow (i.e. sense of challenge and direction), presence (i.e. a sense of belonging) and the way users’ bond with their avatar (i.e. personal narrative [1]). In particular, gaming flow is generated by a system of gradually higher challenges and rewards, embedded within the game-fabric, in a way that follows the pace of increase of a gamer’s skills, maintaining their attention while playing (e.g. if game challenges exceed gamer’s skills, they feel stress and disengage; if gamer’s skills significantly outweigh game challenges, they feel boredom and similarly disengage [1]). Tele-Presence describes the sense of the player that they are not in the offline world but in the world of the game [1]. Game graphics, animation, the responsiveness of the game environment contribute to an immersive experience that fosters a sense of presence, belonging, and shared objectives among users—frequently involving teamwork to achieve collective and individual goals within collaborative frameworks [1]. Additionally, players are often able to customize their in-game character or avatar—including appearance, gender, and equipment—to align with personal preferences [1]. Such customization can facilitate a partial integration of the player’s self-identity with their avatar’s identity, driven by processes such as identification (e.g., perceiving oneself as equivalent to the avatar), immersion (e.g., internalizing the avatar’s in-game responsibilities), and compensation or idealization (e.g., projecting aspirational qualities onto the avatar) [1]. These mechanisms may enhance the player’s sense of accomplishment and enrich their personal narrative as the avatar progresses and achieves within the game world [1]. Furthermore, elements such as presence, flow, and avatar features can be dynamically adjusted through adaptive algorithms designed to personalize gameplay according to the player’s profile, thereby optimizing engagement and playtime [1; 9]. This involves the automated collection and analysis of individual gameplay data to refine the game interface in accordance with user preferences [91].

## Gaming economics (microtransactions and loot-boxes)

In the light of such immersive and player-adaptive gaming features, in-game economics emerged attracting scholar and public attention [92]. Although initially the business model of gaming was rather simple and based on one-off purchases, gradually, and with user participation increasing in multiplayer online gaming, it evolved to subscription-based and later ‘freemium’ games with in-game microtransactions options [92]. Freemium games refer to games that are freely available online with the producers making profit from players who pay for added content, typically of low cost per unit sold, leading to the term ‘microtransaction’ that describes an in-game purchase of gaming content [92]. Microtransactions are one-off purchases of specific gaming content which may be non-functional (cosmetic) or provide a competitive advantage for the player who would otherwise have to spend a lot of time (‘grind’) their way to this advantage [92]. At this point it should be noted that, microtransactions are not limited to freemium games, having first appeared within a subscription-based game (World of Warcraft) in 2009, and have gained an additional layer of complexity with the advent of ‘loot-boxes’ (LBs). LBs refer to game-world elements with unknown definite content, which may be bought by the player via a microtransaction for real currency [92]. Interestingly, the majority of top-grossing mobile games and approximately one third of top-grossing console games contain LBs, with such games being largely available to children aged 12+ [93].

While gaming has, since its early inception, continuously involved winning exotic items and ‘treasure chests’ of unknown content, those were attainable through increased gaming effort rather than immediate purchase [92]. As such, the advent of microtransactions and LBs has raised important questions [92]. Microtransactions which lead to the purchase of content which is definite does increase spending in the game, yet it can be argued that the players can directly assess on their own whether this process is in their interests or not. Purchasing a LB however means that the players do not have a definite pre-determined return on their spending since they may ‘win’ some highly coveted gaming content or even no desirable content at all.

This process of risking an asset in order to potentially gain another asset considered more valuable is akin to actual gambling process and therefore, in-game loot boxes have been suggested to act as a game-monetization feature which promotes risk-taking, gambling-like behaviours, encouraging the potential transition of excessive gamers to pathological gambling behaviours (i.e. addiction replacement or comorbidity [94]). These concerns regarding the process being a pathway to actual gambling appear to be confirmed by recent studies [95]. Indicatively, a prospective cohort study of 2,213 Spanish adolescents aged between 11-17 years followed their progression within six months from purchasing LB to participating in online gambling. Results indicated that online gambling was more prevalent among adolescents who bought LBs at the beginning of the study than among those who did not, with girls 3.5 times more likely to gamble after having bought LBs and boys being twice more likely [95].

Microtransactions in general have also been under close scrutiny for potential amplifying effects on addictive game playing [96]. In particular, a working hypothesis has been put forward, that frequent micro-transactions create a rapid reward-reinforcement response, similar in many respects to what may be observed during problem gambling [96]. However, there are nuances that need to be also considered, regarding peer-pressure to compete, societal pressure through online communities and influential gamers and also the type of microtransaction one is involved with (i.e. whether it is aimed at play-to-win or LB purchases [96]). In light of such developments and hypotheses, more longitudinal and ethnographic studies are required to better clarify the underlying motives and long-term effects of the new business, microtransaction involving models, employed by game developers [96].

## Gaming with (in) social media

Microtransactions and a range of gaming-engagement features have initiated permeate social media, forming new hybrid platforms often referred to as massively multiplayer social media platforms or Social Virtual Reality applications [97]. These platforms primary focus of facilitating social interaction and relationship-building, allowing users to customise and navigate virtual worlds through avatars while interacting with others in real time (e.g., VR Chat [98]). Metaverse—a collective, immersive virtual environment where individuals can communicate, form connections, collaborate, entertain themselves, work, and engage in commerce, frequently involving real-world currencies—is recognised as a broader construct encompassing social VR [98]. Increasingly, established social media platforms are integrating virtual reality and gamification elements, such as avatars, effectively evolving into parallel digital environments (for instance, Facebook’s transformation into Meta [99]). Within these contexts, enhancements through digital twins—virtual replicas of physical objects or processes—as well as financial augmentations, like the exchange of real-life currency for virtual goods and vice versa, serve to deepen user engagement [100]. The full impact of these gamified, social-media-driven transformations remains difficult to assess due to their nascent stage and the ongoing need for greater technological adoption, like more widespread use of VR headsets, to realise the full potential of the Metaverse [101]. Nonetheless, with over 400 million social media users currently engaging with metaverse-related platforms—a figure projected to more than double within the next five years—the importance of understanding disordered gaming behaviours has become increasingly significant [102].

At this point it should be noted that problematic gaming and problematic social media use are considered as distinct though interrelated entities (e.g., it’s been suggested social media use usually entails a higher degree of social interaction and emotional investment compared to gaming) [103]. Indeed, although they have been found to co-occur (with small to medium effect sizes) [104, 105], there is little overlap of individuals who fully meet the symptomatic criteria of both conditions [106]. Thus, a promising line of research has emerged and is devoted to understanding the interaction between problematic gaming and problematic social media usage (hours spent by gamers chatting on social media) [107].

## Gaming for health and education

Within this broader context of gaming expansion, gamified features and mechanics, have started to be incorporated into fields of education and health [108]. Serious games, referring to game-applications that use digital play elements to train or upskill individuals or to support wellbeing, have shown promising results for a proportion of their users [109]. Evidence suggests that immersive serious games may assist with literacy and numeracy training or help moderate depressive and anxious symptoms [108, 109]. Indicatively of the progress in this field, the serious game Endeavor has been the first of its kind to receive Food and Drugs Administration (FDA) clearance to be prescribed by medical doctors to address attention deficit and hyperactivity symptoms in the US, triggering public attention, as well as controversies [110]. Similarly, other serious games, such as Daydream, have been developed with the aim of improving brain activity through personalized neuro-feedback, such as monitoring brain waves to provide in-game signals and responses to enhance users’ control over brain function [111]). Such possibilities are furthered by the concept of digital phenotype [112]. As reduced appetite or sleep may provide the “footprint” (called phenotype) of depression, likewise, one’s bond with their avatar (e.g. identification), when analysed with classification algorithms, can provide the online “footprint” (called digital phenotype) of their mental health, including their risk for disordered gaming. This concept presents an exciting prospect for deciphering the game-cues of flow, presence and the user avatar bond to likely inform diagnostic Serious Games industry.

## Regulating gaming

The growing prevalence and impact of gaming usage worldwide have inevitably prompted the introduction of laws aiming to increase the opportunities and decrease the risks related to it [92]. Such legislative initiatives predominantly address three different domains involving (a) how to support the financial benefits related to the gaming industry; (b) how to decrease the addictive risks related to the excessive usage of games and; (c) finally reducing the risks of individual users presenting with forms of addictive symptoms due to their exposure to specific game features [113–116]. For instance, Australia established the Digital Games Tax Offset (DGTO; Division 378; Income Tax Assessment Act 1997) allowing Australian based game developers to receive a 30% tax deduction for any game development costs occurring nationally, with a view of boosting production and revenue [113]. China on the contrary has introduced much debated legislation emphasizing restricting digital games consumption by minors to the extent that they could only play one hour on Friday, Saturday, Sunday and Legal Holidays [114]. Similarly, The Netherlands (2018) and Belgium (2018) have both voted regulatory laws banning the employment of loot boxes by game publishers on the basis of reinforcing the risk for developing addictive gambling behaviours [115, 116]. Overall, and given the impact of excessive gaming involvement, a portion of experts have been calling for international collaboration regarding more effective gaming regulation regimes [117].

## A tentative research agenda

In conclusion, current literature regarding the concept of Gaming Disorder provided by ICD-11 seems to be a relevant development, but still presents a number of points to be clarified, as clearly emerges from our narrative review. A list of priority points is provided in Table 1.

**Table 1:** Tentative research priorities points

1. Research should focus on better understanding the bio-psycho-social factors affecting gaming involvement, especially in different patient profiles with Gaming Disorder (GD). This is crucial for better diagnosis, treatment, and prevention.
2. GD needs to be differentially diagnosed from other comorbid psychiatric conditions, which can impact its course and treatment outcomes. Further longitudinal studies are required.
3. Identifying biological markers to differentiate individuals with GD is essential for accurate diagnosis.
4. Clear differentiation should be made between GD and problem gambling, especially when in-game purchases are involved. Research should focus on cross-addictive behaviours and vulnerable populations.
5. Research on risk and protective factors for GD is crucial, with a focus on standardizing assessment and developing prevention strategies. Analysing technology use for early detection of problematic gaming is suggested.
6. The gaming industry should play a role in minimizing GD harm through responsible game design. Collaboration between industry and scholars could enhance research translation and improve harm reduction strategies.
7. Further investigation into the most effective treatments for GD is necessary due to varied psychological mechanisms involved. More research on psychological and pharmacological treatments is needed.

Firstly, it is important to clarify the bio-psycho-social and game-specific factors interfering with the different trajectories one may follow in relation to their gaming involvement. For instance, in an attempt to clarify the psychological aspects at the basis of the disorder, a recent study proposed the existence of three main types of patients suffering from GD: impulsive male patients with attention deficit hyperactivity disorder, patients with dysphoria and dysfunctional coping skills and isolated patients with social anxiety [118]. The investigation of psychological mechanisms leading to the development of GD could thus have a relevant impact not only on the diagnosis, but also on treatment choice and prognosis, and should therefore be emphasized as an important area of research. Similarly, although cultural and psychosocial factors are known to play a role in the presentation of psychiatric disorders [13, 14]; research on GD has been rather disproportionately conducted in Asia and Western countries. In this respect, one of the first aspects to be studied in detail is the epidemiology of GD and its possible correlation with country-specific factors world-wide. This is compelling given the globally expansive use of games and therefore, the likely increasing vulnerability of previously game-disorder immune populations and could inform improved prevention initiatives [1, 10, 11].

Secondly, it is the authors opinion that GD should be differentiated from a number of other psychiatric conditions; ICD-11 itself names as potential differential diagnoses mood disorders, anxiety-spectrum disorders, obsessive-compulsive disorder [13, 14]. The importance of this differential diagnosis is increased by the fact that the same conditions could represent comorbidities of GD, and could therefore modify the clinical history of the affected subject. The role of these comorbidities should be more clearly addressed in terms of their impact on the symptomatology, on the course, on the optimal treatment and on the prognosis of GD by further longitudinal empirical evidence and possibly cross-lagged design studies, which enable the identification of any relevant causal effects [1, 119].

Thirdly, and in line with other psychiatric disorders, the identification of neurobiological correlates could substantiate the validity of the Gaming Disorder as a distinct clinical entity. Despite methodological heterogeneity, existing research suggests that individuals with GD exhibit neural and cognitive patterns similar to those observed in gambling and substance-use disorders [120]. Further studies using neuroimaging and cognitive profiling could help distinguish disordered from non-disordered gamers and reduce the risk of both over- and under-diagnosis. For this purpose, available, large cohort, longitudinal datasets, such as the Adolescent Brain and Cognitive Development Dataset (ABCD) data should be used.

Fourthly, a relevant differential diagnosis should be conducted carefully between disordered gaming and problem gambling in the cases where gamers are over-consuming in-game LBs [20]. Given that the majority of high-grossing mobile games include LBs, with clear indications that these are correlated with increased problem gambling risk, the call for their worldwide regulation [96] is compelling. A greater emphasis on cross-addictive or addiction replacement behaviours, their generating mechanisms and vulnerable populations should be in this context provided by future research [96, 113, 121].

Fifthly, a further improved understanding of GD relevant risk and protective factors is required, with research in this respect rather dwarfing the possibilities arising [122]. For instance, a very encouraging and timely paper by Cho et al. [123] explored the possibility to analyse the way technological tools are used in non-gaming activities to predict the development of problematic gaming. Knowing these aspects could be crucial for standardising GD assessment via objective measures and for developing primary prevention strategies meant to increase protective factors and to reduce modifiable risk factors.

Sixthly, the authors support that greater emphasis should be placed on the role and the capacity of the gaming industry to introduce caveats to reduce GD related harm. As the design, production, development, and operation of games typically involve significant costs, it is reasonable for industry stakeholders to anticipate revenue generation through game sales. This objective can be accomplished by ensuring a broad user base and/or by extending player engagement over time, depending on the specific characteristics of the game. However, the developers’ objection to the inclusion of GD in ICD-11 [17, 18] did not align with the existing literature on problematic gaming [20]. It should therefore be investigated, whether and how the industry may adopt an approach that minimizes harm for at-risk players, as expected when a product, such as gaming, has a widespread impact on the life of people all around the world [124]. This could benefit the industry and improve its reputation among current and potential clients. Counter-intuitively, a collaboration between game-disorder scholars and game-developers could significantly boost research translation (e.g. by implementing automated tracking strategies) [20].

Finally, as it is still unclear what treatment has the highest efficacy for those suffering from GD, further investigation is considered essential [121]. The lack of such sufficient knowledge could be due in part to the heterogeneity of the psychological mechanisms involved to the introduction of GD [121, 122], with recent studies also concluding that more research is needed to understand the role of psychological and pharmacological treatments in addressing the condition [125].

All mentioned above points of uncertainty also limit the possibility to design and empirically test preventive interventions; this could be therefore another future direction for research, provided that previously described questions have been answered to the point that a preventive strategy can be designed on sufficiently solid basis.

## Data Availability

Not applicable.

## Abbreviations

APA: American Psychiatric Association
CDF: Cyber-Developmental Framework
DGTO: Digital Games Tax Offset
DSM-5: Diagnostic and Statistical Manual of Mental Disorders – Fifth Edition
F2P: Free-to-play
FDA: Food and Drugs Administration
GD: Gaming Disorder
ICD: International Classification of Diseases
IGD: Internet Gaming Disorder
MMORPG: Massively Multiplayer Role Playing Game
VR: Virtual Reality
WHO: World Health Organization
